# The influence of elastic modulus and compressive strength on the significance of rock failure

**DOI:** 10.1371/journal.pone.0342074

**Published:** 2026-02-26

**Authors:** Yanhong Du, Laigui Wang, Na Zhao, Feng Chen, Kaixing Wang

**Affiliations:** School of Mechanics and Engineering, Liaoning Technical University, Fuxin, Liaoning, China; Sichuan University of Science and Engineering, CHINA

## Abstract

By using numerical simulation methods, the influences of the numerical values of compressive strength and elastic modulus, non-uniformity and the size of heterogeneous regions on the deformation and failure of rocks are analyzed. The research results show that in each group of numerical simulations with different variables, the greater the cumulative acoustic emission (AE) energy, the greater the dilatancy capacity. When the numerical model is destroyed as a whole, the dilatancy capacity rises sharply, and the destruction of the heterogeneous regions of compressive strength and elastic modulus is the reason for the significant increase in the dilatancy capacity of the rock sample. When the overall size of the numerical model is relatively small, the material failure requires a large heterogeneous area of compressive strength, that is, the overall size is the dominant factor. When the overall size of the numerical model is large, a tiny heterogeneous region of compressive strength can lead to overall failure, that is, the local size is the dominant factor. Regardless of whether the numerical model is large or small in size, a smaller elastic modulus heterogeneous region will lead to the failure of the numerical model. The parameters such as the failure mode, dilatancy capacity, AE quantity and AE energy of the numerical model all indicate that under the influence of same external factors, the elastic modulus has a much greater influence on the deformation and failure of rocks than that of the compressive strength.

## 1. Introduction

Rock is a complex geological structure that is heterogeneous, anisotropic, discontinuous and internally stressed. Inside the structure, there are many rock elements with different mechanical properties, and each element itself is often heterogeneous, anisotropic and discontinuous [1–4]. It can be seen from this that the mechanical properties of rocks are far more complex than those of other materials. The failure of rocks is mainly caused by the heterogeneity of their mechanical properties, which includes the heterogeneity of compressive strength and elastic modulus [5–7]. The interior of rocks contains heterogeneous bodies, and the size of the heterogeneous regions of compressive strength and elastic modulus affects the deformation and failure of rocks.

The heterogeneity of rocks has a significant impact on the macroscopic strength and nonlinear deformation behavior of rock samples, and also significantly affects the fracture mode of the specimens. Many scholars have studied the influence of the heterogeneity of rocks on their fracture morphology [8–14]. The manifestations of rock heterogeneity include the following four types [15–23]: ① Physical properties: spatial variations in density, porosity and permeability. ② Mechanical properties: local differences in elastic modulus, compressive strength, tensile strength and fracture toughness. ③ Structural features: the randomness of fracture network, bedding planes and mineral particle distribution. ④ Anisotropy: The difference in properties in different directions (such as the strength difference between parallel and vertical bedding directions of shale). Among them, the elastic modulus of rock is an important parameter characterizing the elastic deformation capacity of rock under force, and it is also one of the core parameters in rock mechanics analysis, reflecting the rock’s ability to resist elastic deformation. Rock compressive strength refers to the ability of rocks to resist failure under uniaxial compressive loads, it is an important parameter for evaluating the mechanical properties of rocks in engineering geology and geotechnical engineering. It directly affects the feasibility and safety of projects such as foundation bearing capacity, tunnel support design, slope stability analysis and mining. In view of the important role of rock elastic modulus and compressive strength in rock mechanics analysis, this paper focuses on the analysis of the influence of the heterogeneity of the two on the deformation and failure of rock.

## 2. Numerical model

Since the 2D RFPA basic version software takes into account the heterogeneity of rocks, this software is used to analyze the influence of the magnitudes of compressive strength and elastic modulus, heterogeneity and the size of heterogeneous regions on the deformation and failure characteristics of rocks. Calculate the compressive strength and elastic modulus corresponding to different degrees of homogeneity *m* by using Equations (1) and (2). When *m* = 30, the numerical model is regarded as a homogeneous body, the compressive strength and elastic modulus adopted in the numerical simulation are the same as those obtained from the laboratory test, that is, the mesoscopic values of compressive strength and elastic modulus are equal to the macroscopic values. When *m* is 3 and 1.5 respectively, the numerical model is regarded as a heterogeneous body. The mechanical parameters of the heterogeneous body are shown in Table 1, and the mesoscopic values of compressive strength and elastic modulus are not equal to the macroscopic values. The macroscopic values of each mechanical parameter are obtained through the uniaxial compression chamber test, and the mesoscopic values of each mechanical parameter are the input values of the numerical simulation. The difference in homogeneity only causes the difference in $f^{\prime }$ and $E^{\prime }$, does not affect other physical and mechanical parameters.

**Table 1:** $E^{\prime }$ and $f^{\prime }$ with different homogeneity degrees.

*m*	$f^{\prime }$ (MPa)	$E^{\prime }$ (MPa)
30	20.69	3.28
3	66.96	4.09
1.5	159.15	4.65


$$\frac{f^{\prime \prime }}{f^{\prime }}=0.2602\;ln\;m+0.0233(1.2\leq m\leq 50)$$ (1)

$$\frac{E^{\prime \prime }}{E^{\prime }}=0.1412\;ln\;m+0.6476(1.2\leq m\leq 50)$$ (2)

In the formula: $E^{\prime }$ and $f^{\prime }$ represent the elastic modulus and uniaxial compressive strength of the numerical simulation input respectively; $E^{\prime \prime }$ and $f^{\prime \prime }$ are the elastic modulus and uniaxial compressive strength obtained from the uniaxial compression test, respectively. The *m* is the uniformity coefficient of the rock, and its physical meaning reflects the uniformity of the rock medium. The larger *m* is, the better the degree of uniformity of the rock.

The size of each cell is guaranteed to be 0.5 mm in any numerical model. The uniaxial compression numerical simulation is conducted on all numerical models, with a load increment of 0.005 mm. For the convenience of description, the symbol 20 (40, 60,...)-2 (4, 6,...)-SHe (or EHe) is used to represent the numerical model. Among them, 20 (30, 40,...) represents the number of rows and columns of the square numerical model is 20 (30, 40,...), and the side length of the square numerical model is 10 mm (15 mm, 20 mm,...); 2 (4, 6,...) represents the change of the mechanical properties of 2 rows and 2 columns (4 rows and 4 columns, 6 rows and 6 columns, etc.) elements at the center of the numerical model, which is different from the mechanical properties of the overall numerical model. SHe represents compressive strength heterogeneity, and EHe represents elastic modulus heterogeneity. For instance, the specific meaning of 20–2-SHE is that the size of the numerical model is 10 × 10 mm, the number of divided elements is 20 × 20, and the compressive strength of the two rows and two columns of elements in the middle of the numerical model is heterogeneous, while the elastic modulus is homogeneous. The meanings of the shorthand symbols for other numerical models can be inferred in the same way. The axial strain and lateral strain of the middle element on the right side of the numerical model are taken to calculate the volumetric strain, and the stress-volumetric strain curve is used to analyze the dilatancy characteristics of the rock.

## 3. The influence of compressive strength differences on the deformation and failure characteristics of rocks

### 3.1. The size of the numerical model remains unchanged, while the heterogeneous region gradually increases

**Fig 1:**
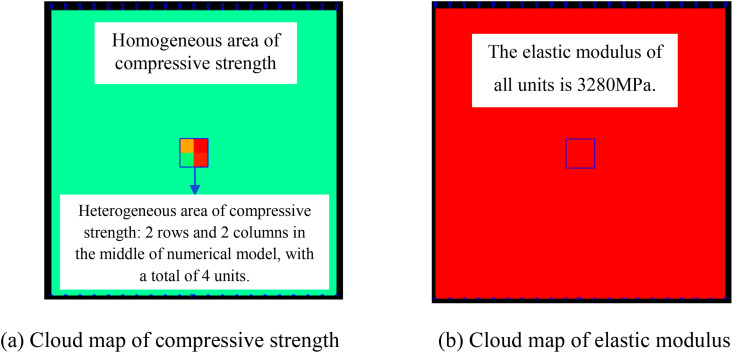
Numerical model of heterogeneous compressive strength and homogeneous elastic modulus.

To analyze the influence of the size of the heterogeneous region of compressive strength on the deformation and failure characteristics of rocks, ten numerical models are established. The overall size of all numerical models is 10 × 10 mm, and the number of units divided is all 20 × 20.The compressive strength of the elements in the middle region of the numerical model is heterogeneous and the elastic modulus is homogeneous, while the compressive strength and elastic modulus in the remaining regions are homogeneous, as shown in Fig 1. The homogeneity degree *m* in the heterogeneous area is 3.

**Fig 2:**
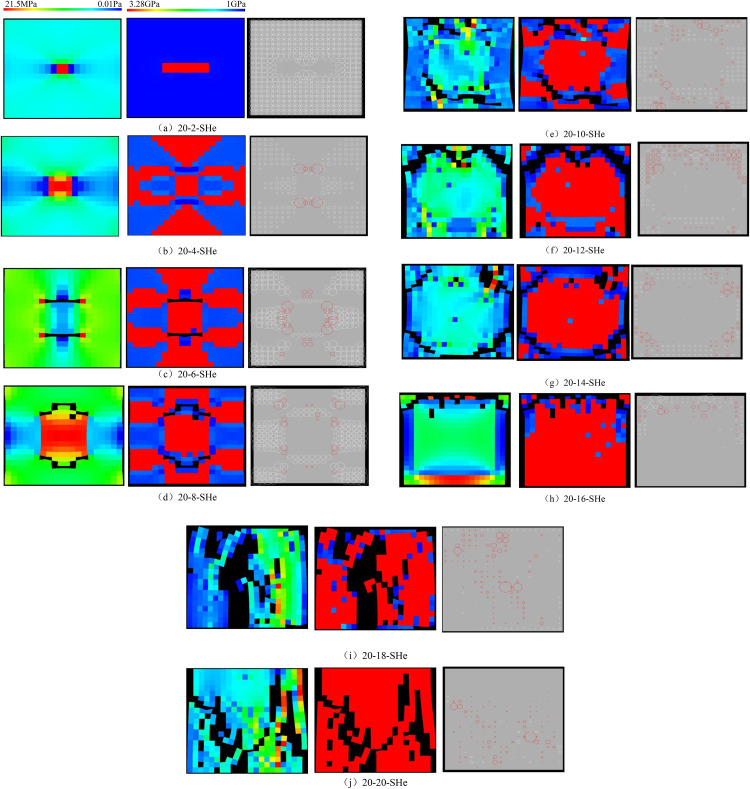
Deformation and failure modes of different numerical models, the left side is the maximum principal stress cloud map, the middle is the elastic modulus cloud map, and the right side is the AE cloud map.

**Fig 3:**
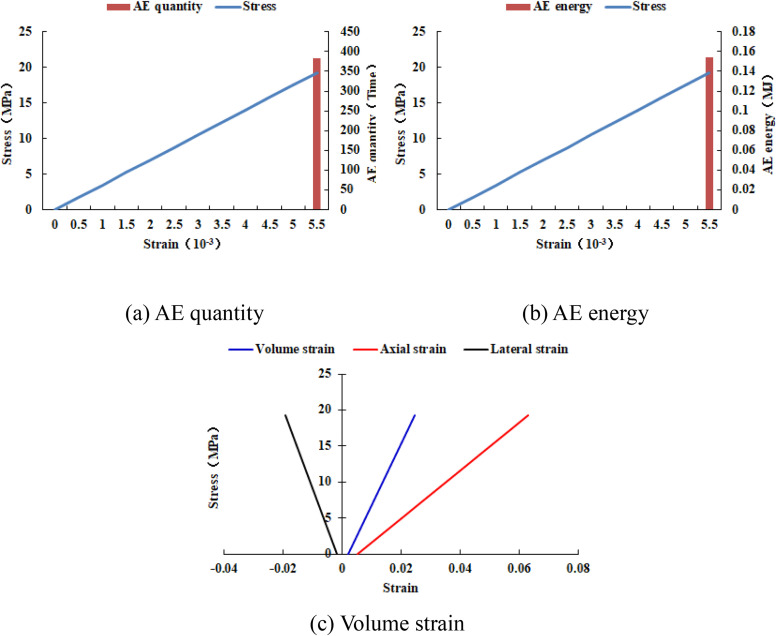
Variation trends of AE quantity, AE energy and volume strain of numerical model 20-2-SHe.

When the heterogeneous region of compressive strength is in 2 rows and 2 columns, that is, the side length of the heterogeneous region is one-tenth of the numerical model (as shown in Fig 2(a)), it can be known from the AE cloud map that at the last moment of loading, all elements in the homogeneous region of the numerical model undergo shear deformation, but no damage occurs, and the heterogeneous region of compressive strength in the model does not deform. It can be seen from the elastic modulus cloud map that the elastic modulus of the units without AE is all 3280 MPa. It can be known from the maximum principal stress cloud map that the maximum principal stress values of the elements in the heterogeneous region in the middle of the numerical model are the same, while the maximum principal stress values of the elements close to the heterogeneous region in the homogeneous region are different. Gradually moving away from the heterogeneous region, the maximum principal stress values of the units in the homogeneous region tend to be consistent again, and their values are lower than those in the heterogeneous region. From this, it can be known that the heterogeneity of rocks is the cause of the increase in unit stress. From the variation curves of AE quantity and AE energy (see Fig 3), it can be seen that only at the last step of loading, the unit undergoes sudden deformation and releases energy, and the stress endured by the numerical model almost reaches the compressive strength of the material. The stress-lateral strain curve, stress-axial strain curve and stress-volumetric strain curve only have the linear elastic stage and no post-peak stage, the numerical model has not undergone dilatancy, and the dilatancy capacity is 0.

**Fig 4:**
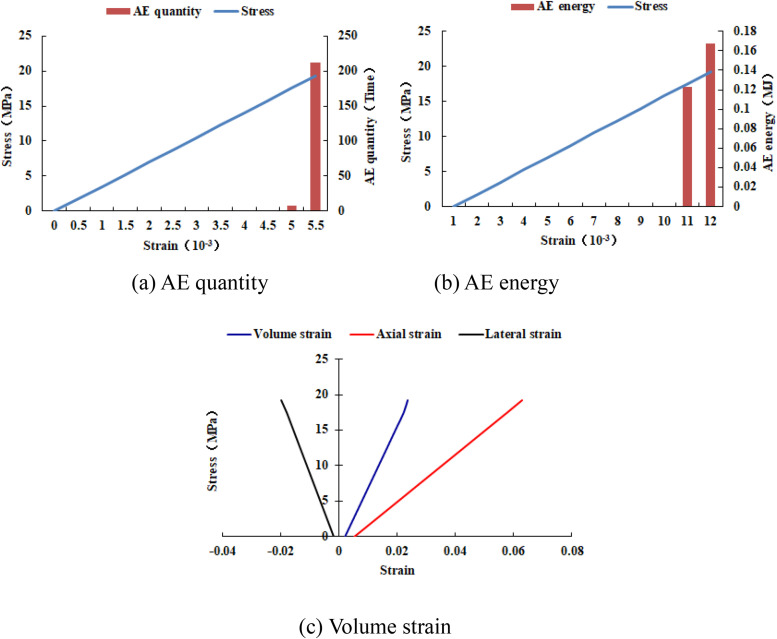
Variation trends of AE quantity, AE energy and volume strain of numerical model 20-4-SHe.

When the heterogeneous region of compressive strength is in 4 rows and 4 columns, that is, the side length of the heterogeneous region is two-fifths of the numerical model (as shown in Fig 2(b)), It can be known from the AE cloud map that when the loading reached the last moment, some elements in the homogeneous region of the numerical model occur symmetrical shear deformation, while the upper and lower boundary elements in the heterogeneous region occur tensile deformation. However, all elements are not damaged, and the inner boundary elements in the heterogeneous region are not deformed. By combining the AE cloud map and the elastic modulus cloud map, it can be known that the elastic modulus of the element without deformation is 3280 MPa. It can be seen from the maximum principal stress cloud diagram that the maximum principal stress of the heterogeneous region element in the middle of the numerical model gradually decreases from the inside to the outside. It can be seen from the AE quantity curve graph (see Fig 4) that a large number of elements deform at the final step of loading. There is no energy release in the early stage of the numerical model, and only in the last two steps of loading does the numerical model have energy release. The stress endured by the numerical model almost reaches the compressive strength of the material. The stress-lateral strain curve, stress-axial strain curve and stress-volumetric strain curve only have the linear elastic stage and no post-peak stage, the numerical model has not undergone dilatancy.

**Fig 5:**
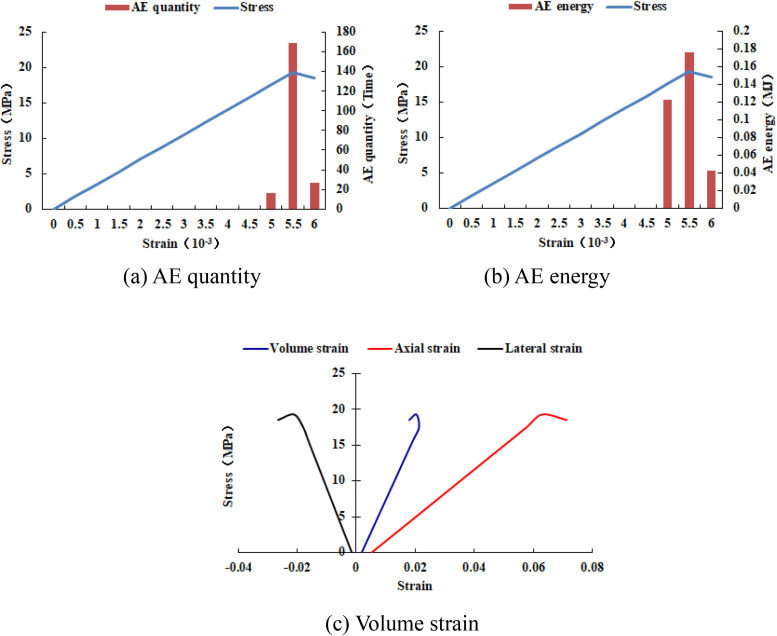
Variation trends of AE quantity, AE energy and volume strain of numerical model 20-6-SHe.

**Fig 6:**
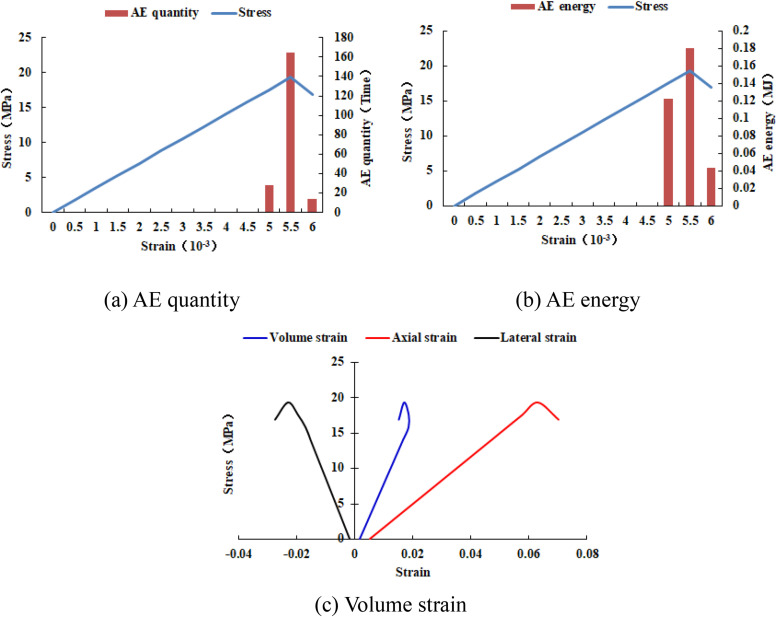
Variation trends of AE quantity, AE energy and volume strain of numerical model 20-8-SHe.

**Fig 7:**
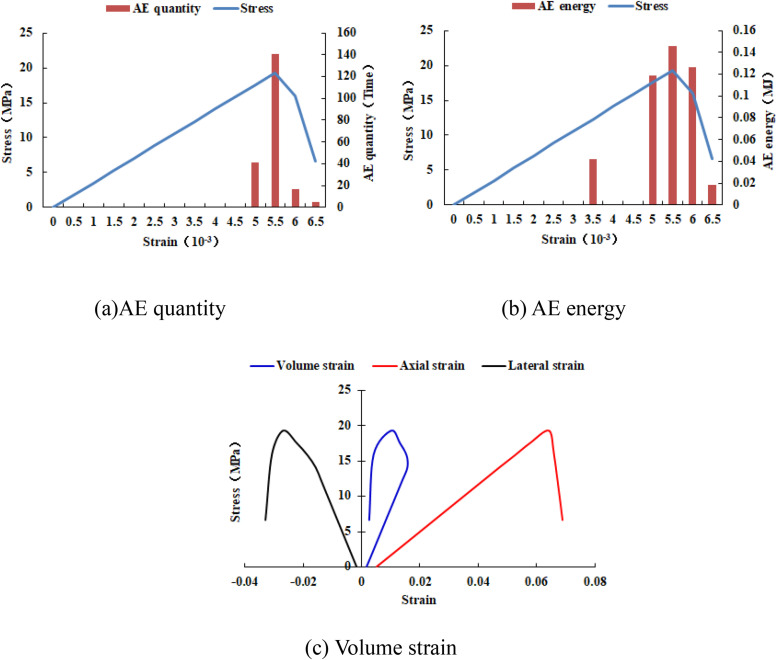
Variation trends of AE quantity, AE energy and volume strain of numerical model 20-10-SHe.

**Fig 8:**
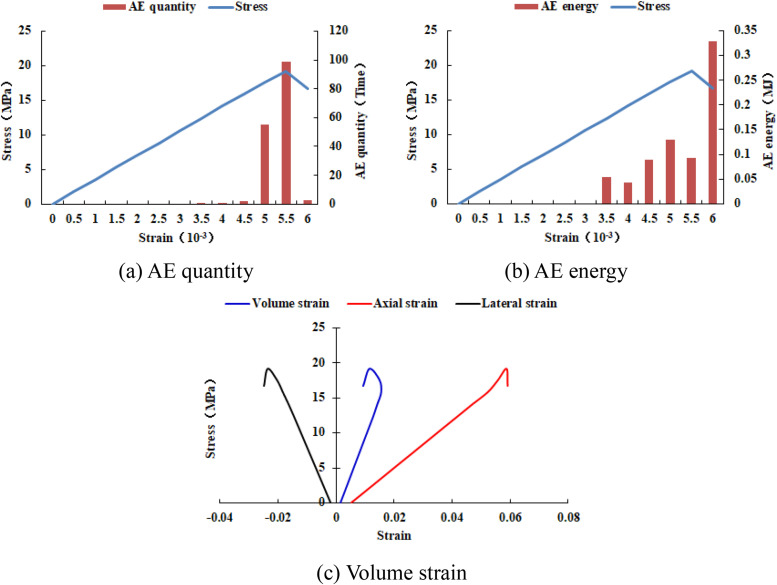
Variation trends of AE quantity, AE energy and volume strain of numerical model 20-12-SHe.

**Fig 9:**
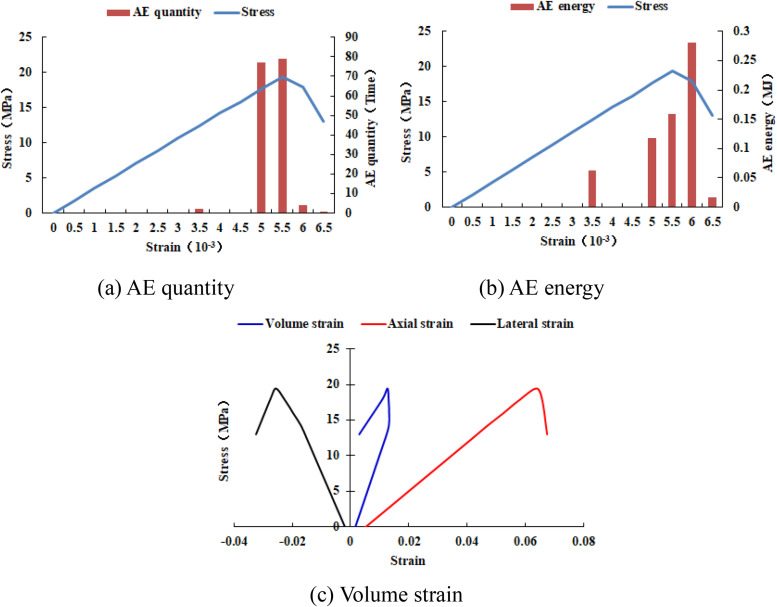
Variation trends of AE quantity, AE energy and volume strain of numerical model 20-14-SHe.

**Fig 10:**
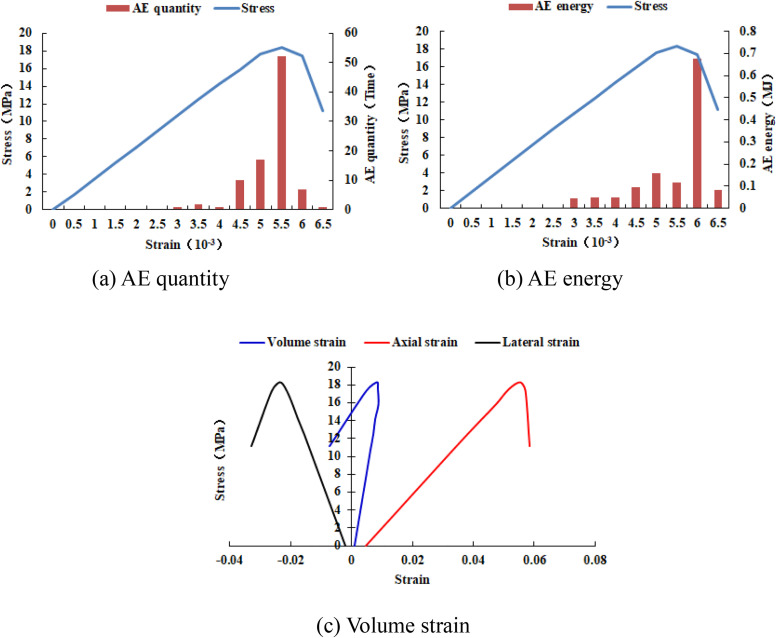
Variation trends of AE quantity, AE energy and volume strain of numerical model 20-16-SHe.

**Fig 11:**
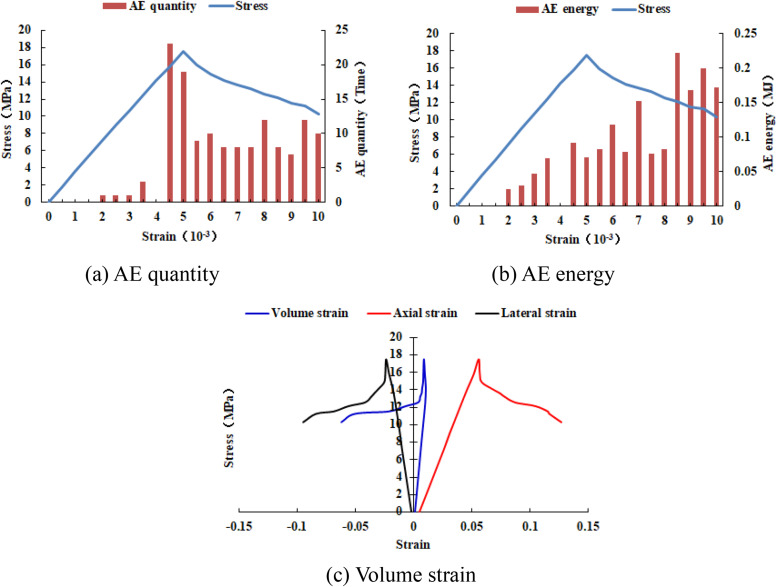
Variation trends of AE quantity, AE energy and volume strain of numerical model 20-18-SHe.

**Fig 12:**
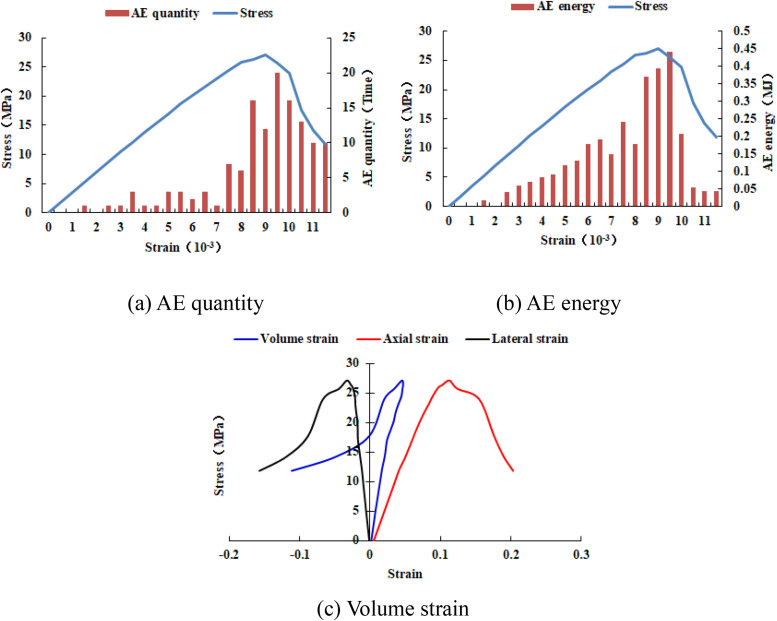
Variation trends of AE quantity, AE energy and volume strain of numerical model 20-20-SHe.

It can be seen from Figs 2(c)–2(h) that with the increase of the heterogeneous region of compressive strength, the failure range of the homogeneous region in the numerical model gradually expands. From the AE diagram, it can be seen that the damaged element has tensile deformation. The compressive strength heterogeneous region remains undamaged, and the elastic modulus is 3280 MPa. The stress-lateral strain curve and the stress-axial strain curve have pre-peak and post-peak stages. It can be seen from Figs 3–12 that both the AE quantity and the stress reach their maximum values simultaneously. Moreover, only the last 3–4 loading steps numerical model generates AE. When the number of rows in the compressive strength heterogeneous region increases from 2 rows to 16 rows, the occurrence time of the maximum AE energy shifts from the peak strain stage to the post-peak stage. It can be seen from the AE quantity and AE energy curves that both suddenly reach their maximum values, indicating that the numerical model has brittle deformation failure.

**Fig 13:**
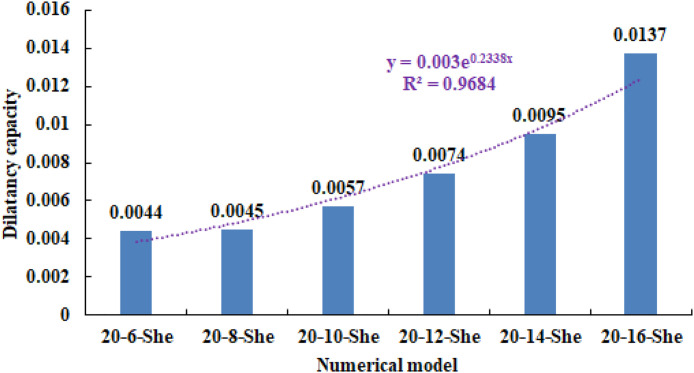
Variation trend of dilatancy capacity in different numerical models.

As can be seen from Table 2, when the number of rows in the compressive strength heterogeneous region increases from 6 rows to 16 rows, the dilatancy capacity increases from 0.0044 to 0.0137. The stress-volume strain curve has a linear elastic stage and an accelerated dilatancy stage. The dilatancy capacity of the numerical model increases exponentially with the linear increase of the heterogeneous region of compressive strength, as shown in Fig 13. When the number of rows in the heterogeneous region of compressive strength increased to 18 rows and 20 rows respectively (the compressive strength of overall numerical model is heterogeneous), obvious tensile failure occurred in the heterogeneous region of compressive strength of the numerical model (see Figs 2(i) and 2(j)), and the dilatancy capacity of the numerical model increased significantly, with the values of 0.0706 and 0.1287 respectively. Compared with the dilatancy capacity of the numerical model that is not destroyed in the first 8 kinds of compressive strength heterogeneous regions, the overall failure of the numerical model is the reason for the rapid growth of the dilatancy capacity.

**Table 2:** The dilatancy capacity, compressive strength, AE quantity and AE energy of different numerical models when the side length of the numerical model is 10 mm.

Numerical model	Dilatancy capacity	Compressive strength (MPa)	Maximum value of AE quantity (Time)	Cumulative AE quantity (Time)	Maximum value of AE energy (MJ)	Cumulative AE energy (MJ)
20-2-SHe	0	19.25	384	384	0.15	0.15
20-4-SHe	0	19.27	212	220	0.17	0.29
20-6-SHe	0.0044	19.30	169	212	0.18	0.34
20-8-SHe	0.0045	19.34	165	207	0.18	0.35
20-10-SHe	0.0057	19.33	141	205	0.15	0.45
20-12-SHe	0.0074	19.19	99	161	0.33	0.74
20-14-SHe	0.0095	19.40	79	163	0.28	0.64
20-16-SHe	0.0137	18.34	52	91	0.68	1.27
20-18-SHe	0.0706	17.54	23	140	0.22	1.68
20-20-SHe	0.1287	27.07	20	130	0.44	3.1

**Fig 14:**
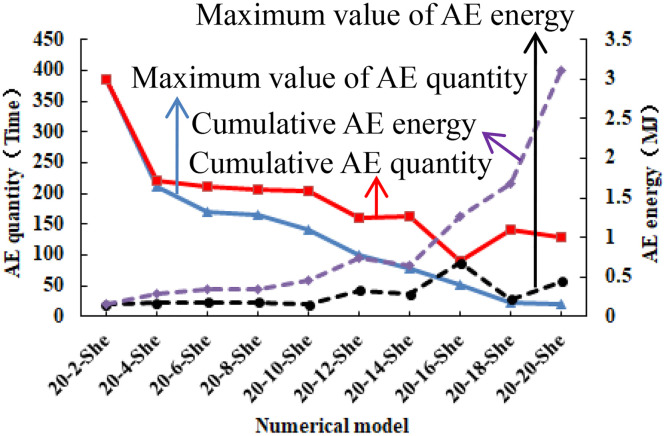
Variation trend of AE in different numerical models.

The distribution of AE quantity and AE energy is in the whole loading stage, and the distribution pattern of the two is very different from that of the previous numerical model. As the number of elements in the heterogeneous region of compressive strength increases, the maximum value of AE quantity and the cumulative number of AE gradually decrease, and the maximum value of AE energy and the cumulative AE energy gradually increase (see Fig 14). It can be seen from Table 2 that when the number of rows in the heterogeneous region of compressive strength increases from 2 to 20, the maximum value of AE quantity decreases from 384 to 20, the cumulative number of AE decreases from 384 to 130, the maximum value of AE energy increases from 0.15 MJ to 0.44 MJ, and the cumulative AE energy increases from 0.15 MJ to 3.1 MJ. The above phenomena indicate that the heterogeneous region of compressive strength is not easy to be damaged, but the energy released when it is damaged is large, the homogeneous region of compressive strength is easy to be destroyed, but less energy is released when it is damaged.

### 3.2. The influence range of the heterogeneous region of compressive strength

From the analysis in the previous section, it can be known that when the size of the numerical model is 0.01 × 0.01 m and the heterogeneous area of the compressive strength is 2 rows and 2 columns, the numerical model does not occur deform and fail. To analyze the influence range of the heterogeneous region of compressive strength, it is assumed that the size of the heterogeneous region of compressive strength remains unchanged, and the numerical model gradually increases, which are 0.015 × 0.015 m (30 rows and 30 columns), 0.02 × 0.02 m (40 rows and 40 columns), 0.025 × 0.025 m (50 rows and 50 columns), and 0.03 × 0.03 (60 rows and 60 columns).

**Fig 15:**
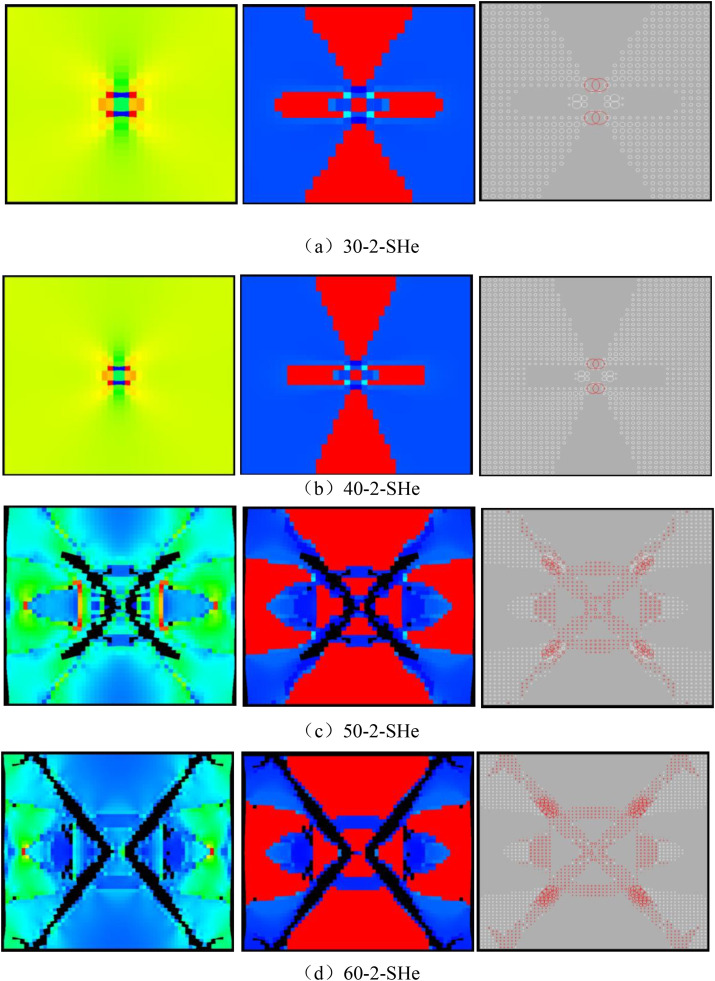
Deformation and failure modes of different numerical models.

**Fig 16:**
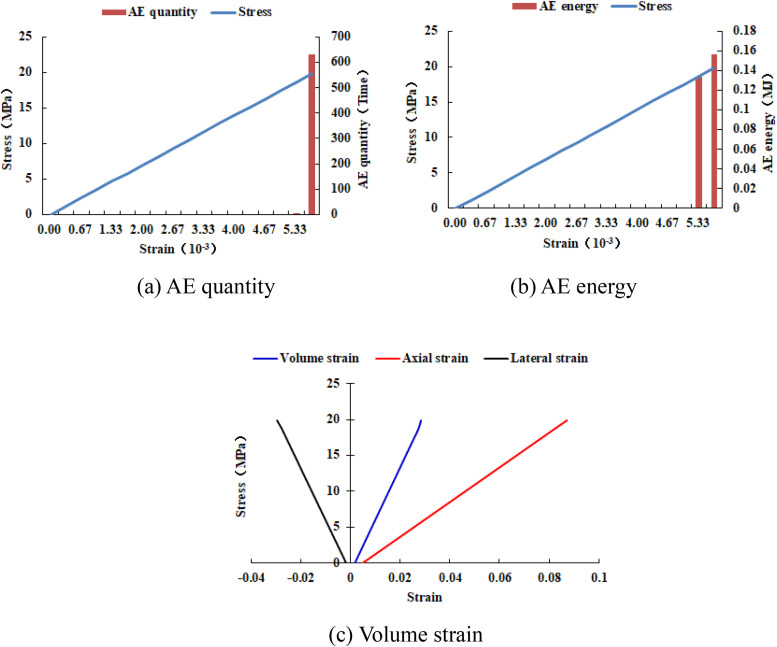
Variation trends of AE quantity, AE energy and volume strain of numerical model 30-2-SHe.

**Fig 17:**
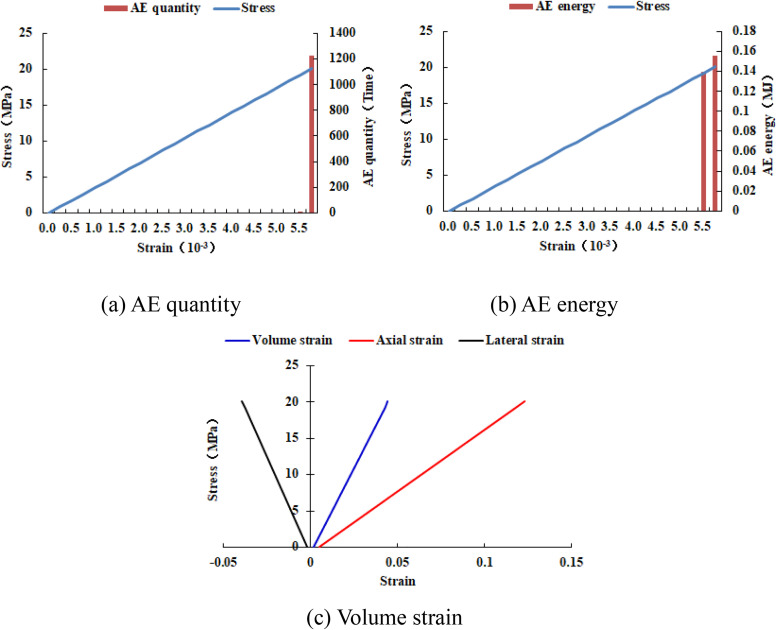
Variation trends of AE quantity, AE energy and volume strain of numerical model 40-2-SHe.

**Fig 18:**
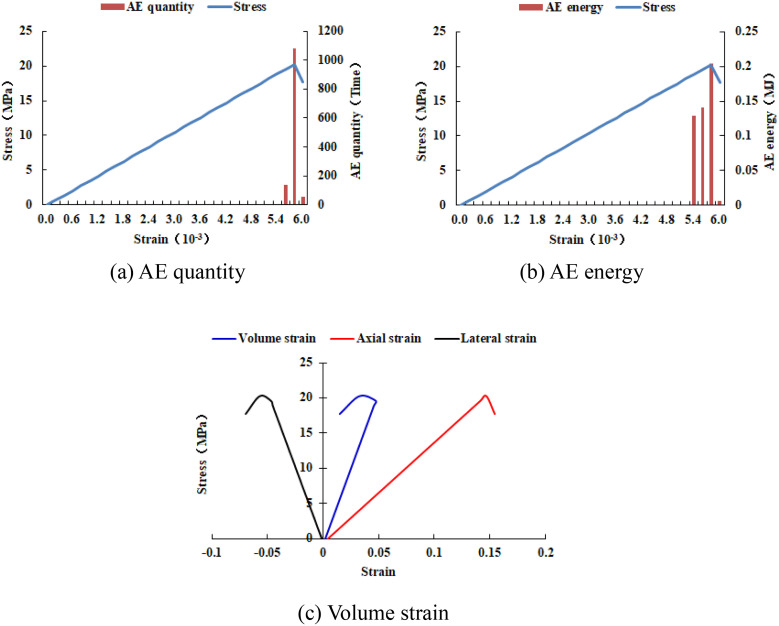
Variation trends of AE quantity, AE energy and volume strain of numerical model 50-2-SHe.

**Fig 19:**
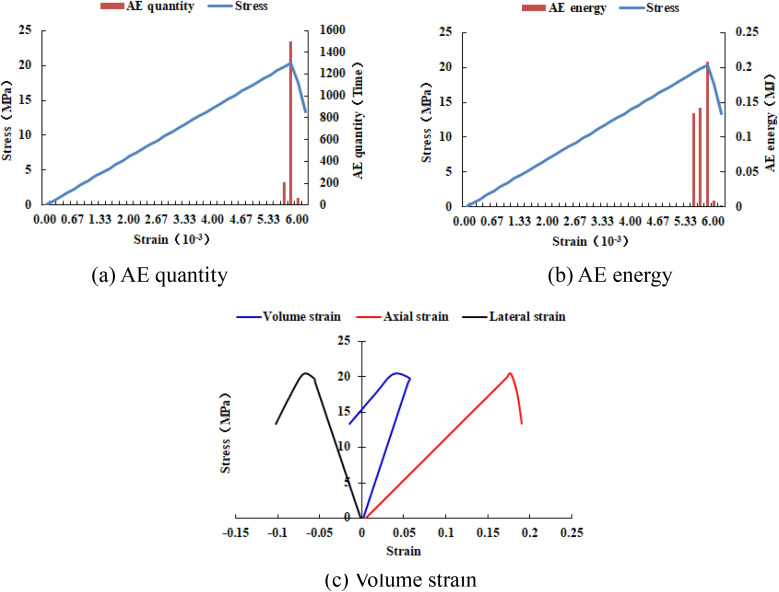
Variation trends of AE quantity, AE energy and volume strain of numerical model 60-2-SHe.

As can be seen from Fig 15, when the number of rows of the numerical model increases to 40, the numerical model is not damaged and the dilatancy capacity is 0. When the number of rows of the numerical model increases to 50, the ‘X-shaped’ fracture zone appears in the numerical model, but it does not penetrate the upper and lower boundaries, and the dilatancy capacity is 0.028 (see Table 3). When the number of rows of the numerical model increases to 60, the ‘X-shaped’ fracture zone runs through the upper and lower boundaries, and the dilatancy capacity is 0.054. As shown in Figs 16–19, the maximum value of AE quantity and AE energy of all numerical models occur at the peak strain. At this moment, the AE quantity is much greater than at other times, that is, a large number of elements of the numerical model have suddenly been damaged. However, the AE energy does not suddenly reach its maximum value. Before reaching the maximum value, the numerical model has already released the AE energy, and the AE energy released at different times is of the same order of magnitude.

**Table 3:** The dilatancy capacity, compressive strength, AE quantity and AE energy of each numerical model with the size of the heterogeneous region of compressive strength remaining unchanged (0.001*0.001 m) and the overall numerical model gradually increasing.

Numerical model	Dilatancy capacity	Compressive strength (MPa)	Maximum value of AE quantity (Time)	Cumulative AE quantity (Time)	Maximum value of AE energy (MJ)	Cumulative AE energy (MJ)
30-2-SHe	0	19.83	632	636	0.16	0.29
40-2-SHe	0	20.12	1224	1228	0.16	0.3
50-2-SHe	0.028	20.22	1082	1282	0.2	0.48
60-2-SHe	0.054	20.36	1498	1781	0.21	0.49

The compressive strength of all numerical models is basically the same, approximately 20 MPa. As the overall numerical model increases, the maximum value of AE quantity and the cumulative AE quantity also increase accordingly. However, the maximum value of AE energy and the cumulative AE energy of the numerical models 30–2-SHe and 40–2-SHe are basically the same, and those of the numerical models 50–2-SHe and 60–2-SHe are basically the same.

Table 4 lists the sizes of the heterogeneous regions of compressive strength that lead to the overall failure of the numerical model. It can be seen from this table that when the overall size of the numerical model is relatively small, the material failure requires a large compressive strength heterogeneous region, indicating that the overall size is the dominant factor. When the overall size is relatively large, a tiny heterogeneous area of compressive strength can lead to overall failure, indicating that local size is the dominant factor.

**Table 4:** The size of the heterogeneous region of compressive strength that leads to the overall failure of the numerical model.

Numerical model	Overall size of the numerical model (mm)	The overall number of elements of the numerical model	The size of the heterogeneous zone of compressive strength (mm)	The number of elements in the heterogeneous region of compressive strength
20-18-SHe	0.01*0.01	20*20	0.009*0.009	18*18
30-10-SHe	0.015*0.015	30*30	0.005*0.005	10*10
40-4-SHe	0.02*0.02	40*40	0.002*0.002	4*4
50-4-SHe	0.025*0.025	50*50	0.002*0.002	4*4
60-2-SHe	0.03*0.03	60*60	0.001*0.001	2*2

### 3.3. Heterogeneity of compressive strength

Since the numerical model 20–2-SHe did not undergo dilatancy or damage, this numerical model is used to analyze the influence of the heterogeneity of compressive strength on the deformation and failure characteristics of rocks. The homogeneity of compressive strength is taken as 1.5, and at this time, *f’* = 159.15 MPa.

**Fig 20:**
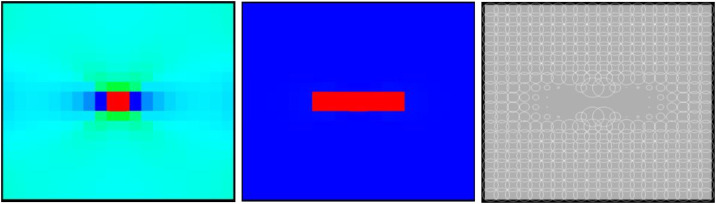
Deformation form of numerical model.

**Fig 21:**
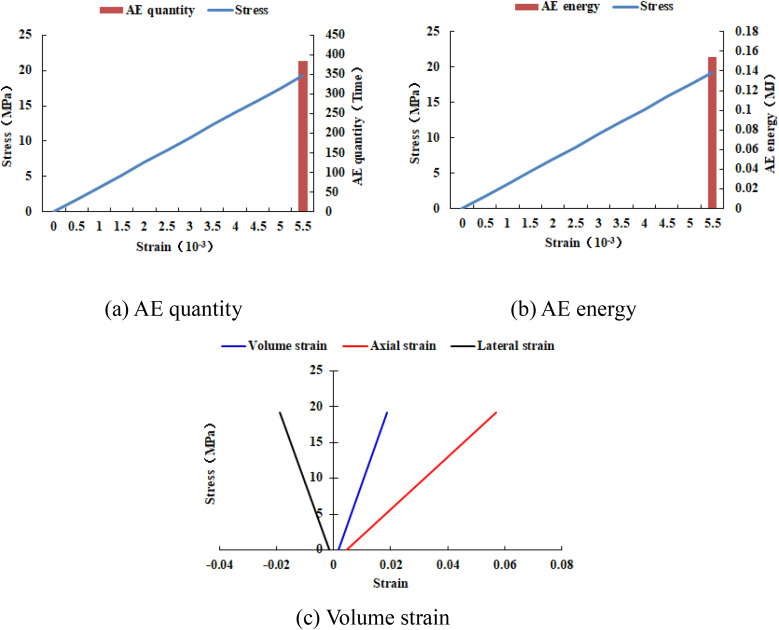
Variation trends of AE quantity, AE energy and volume strain of numerical model 20-2-EHe with a homogeneity of 1.5.

By comparing Fig 2(a) with Fig 20, and Fig 3 with Fig 21, it can be seen that when the homogeneity of compressive strength decreases from 3 to 1.5, the deformation form and AE mode of the numerical model remain unchanged, and the changing trends and values of AE quantity, AE energy and volumetric strain also remain unchanged. From this, it can be known that the heterogeneity of compressive strength has a negligible influence on the deformation and failure of rocks with a relatively small heterogeneous area of compressive strength.

## 4. The influence of elastic modulus differences on the deformation and failure characteristics of rocks

### 4.1. The size of the numerical model remains unchanged, while the heterogeneous region gradually increases

**Fig 22:**
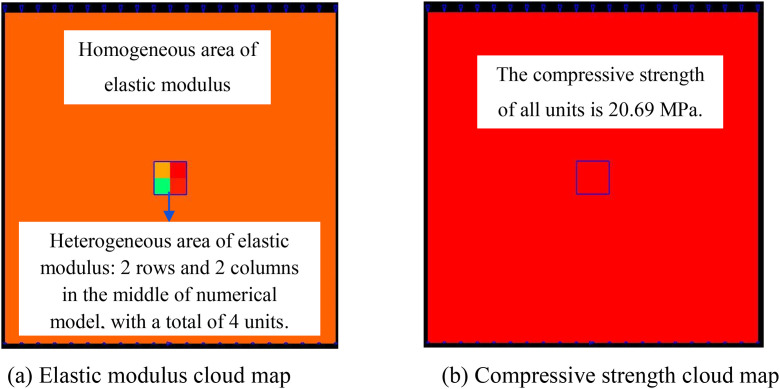
Numerical models of heterogeneous compressive strength and homogeneous elastic modulus.

The overall size of all numerical models is 10 mm × 10 mm, and the number of elements divided is all 20 × 20. The compressive strength of the elements in the middle region of the numerical model is homogeneous, while the elastic modulus is heterogeneous, and the compressive strength and elastic modulus in the remaining regions are homogeneous, as shown in Fig 22. The homogeneity degree m in the heterogeneous region is 3.

**Fig 23:**
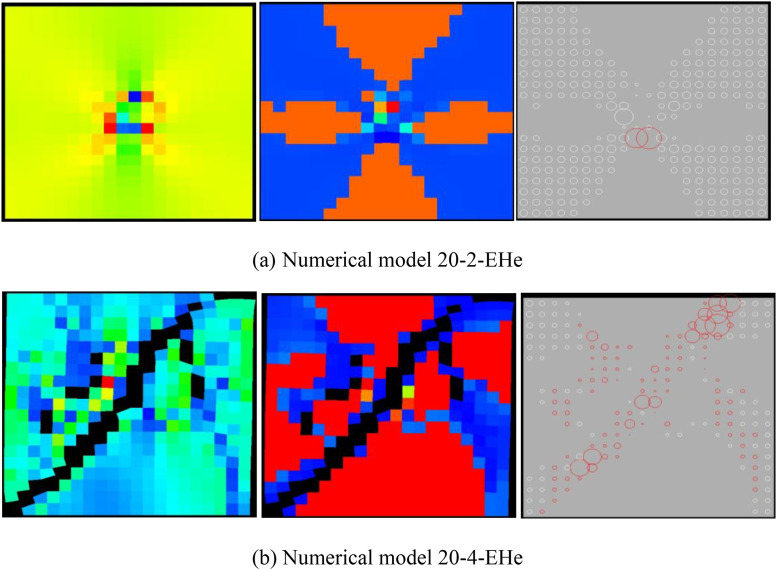
Failure Modes of different numerical models.

**Fig 24:**
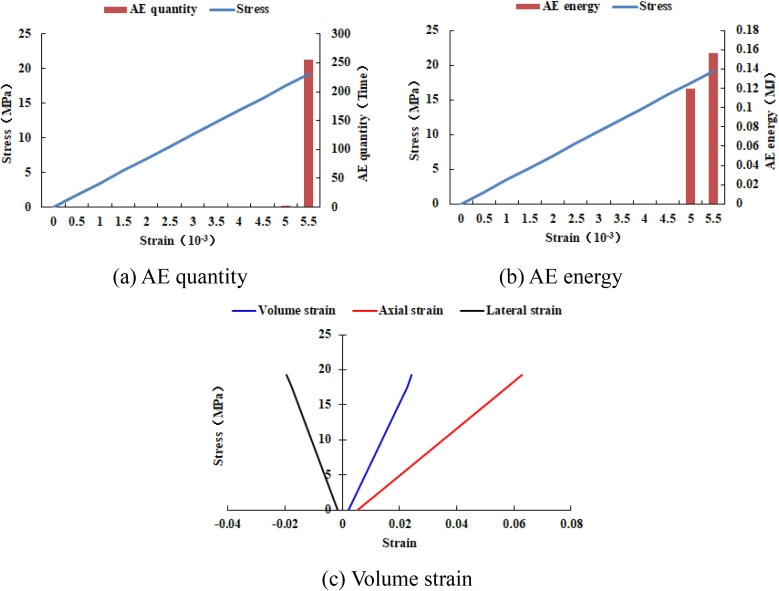
Variation trends of AE quantity, AE energy and volume strain of numerical model 20-2-EHe.

**Fig 25:**
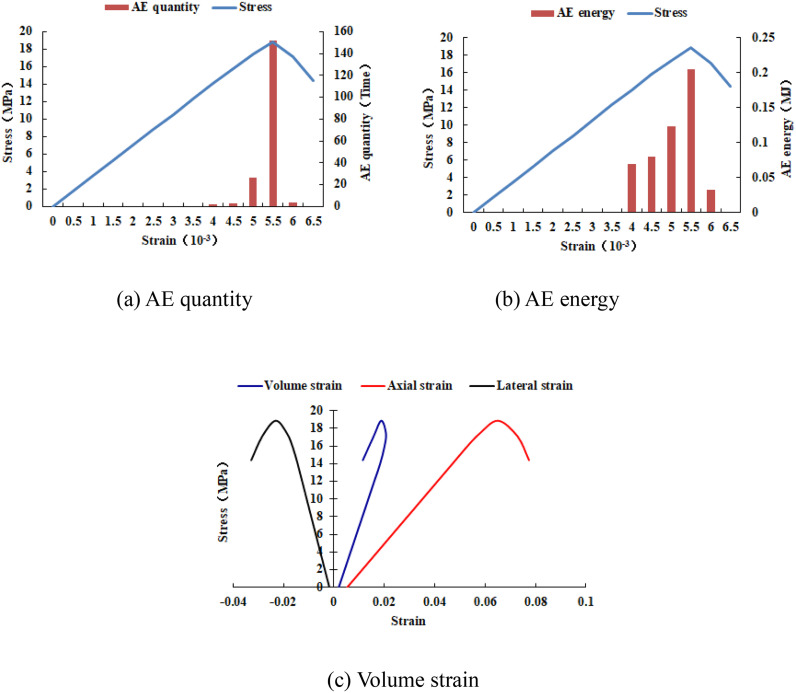
Variation trends of AE quantity, AE energy and volume strain of numerical model 20-4-EHe.

When the heterogeneous region of the elastic modulus is 2 rows and 2 columns, the numerical model is not damaged, some elements have shear deformation, and only 2 elements have tensile deformation (see Fig 23(a)). The stress-strain curve only has the linear elastic stage, and the dilatancy capacity of the numerical model is 0 (see Table 5). When the heterogeneous region of the elastic modulus is 4 rows and 4 columns, the entire numerical model is damaged. It can be known from the AE cloud map that the elements on the failure zone have tensile deformation. It can be seen from the elastic modulus cloud map that the element with a lower elastic modulus has shear deformation (see Fig 23(b)). The stress-strain curve has pre-peak and post-peak stages, the dilatancy capacity of the numerical model is 0.007, which is equivalent to the dilatancy capacity of the numerical model 20–12-SHe. It can be seen from Figs 24 and 25 that maximum value of AE quantity and AE energy of both numerical models occur at the peak strain. With the increase of the heterogeneous region of the elastic modulus, the maximum value of AE quantity decreases from 255 to 152, the cumulative AE quantity decreases from 258 to 187, the maximum value of AE energy increases from 0.16 MJ to 0.21 MJ, and the cumulative AE energy increases from 0.26 MJ to 0.51 MJ. The cumulative AE energy of the numerical model 20–4-EHe is approximately twice that of the numerical model 20–2-EHe.

**Table 5:** The dilatancy capacity, compressive strength, AE quantity and AE energy of the numerical model in heterogeneous regions with different elastic moduli when the side length of the numerical model is 0.01 m.

Numerical model	Dilatancy capacity	Compressive strength (MPa)	Maximum value of AE quantity (Time)	Cumulative AE quantity (Time)	Maximum value of AE energy (MJ)	Cumulative AE energy (MJ)
20-2-EHe	0	19.25	255	258	0.16	0.26
20-4-EHe	0.007	18.87	152	187	0.21	0.51

### 4.2. The influence range of elastic modulus heterogeneous region

**Fig 26:**
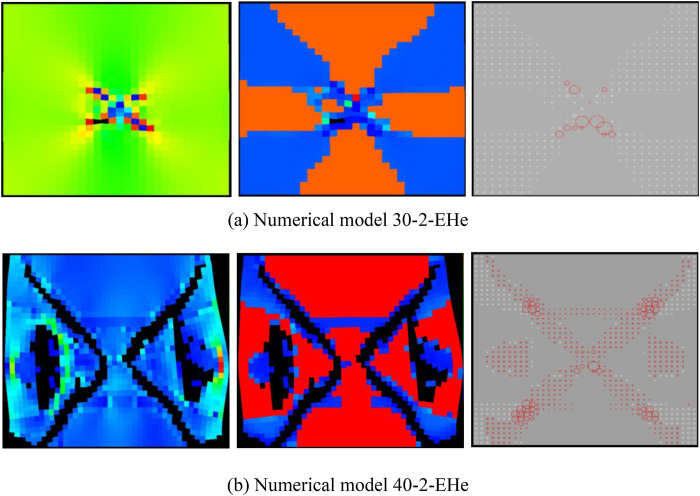
Failure Modes of different numerical models.

**Fig 27:**
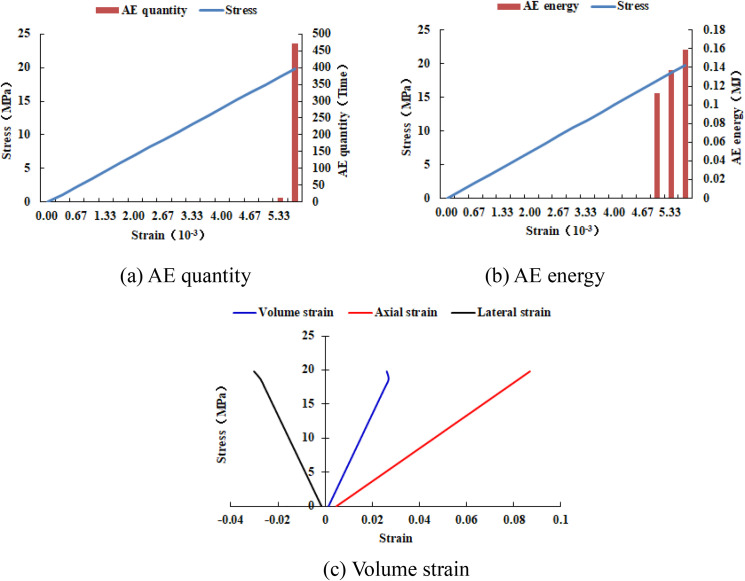
Variation trends of AE quantity, AE energy and volume strain of numerical model 30-2-EHe.

**Fig 28:**
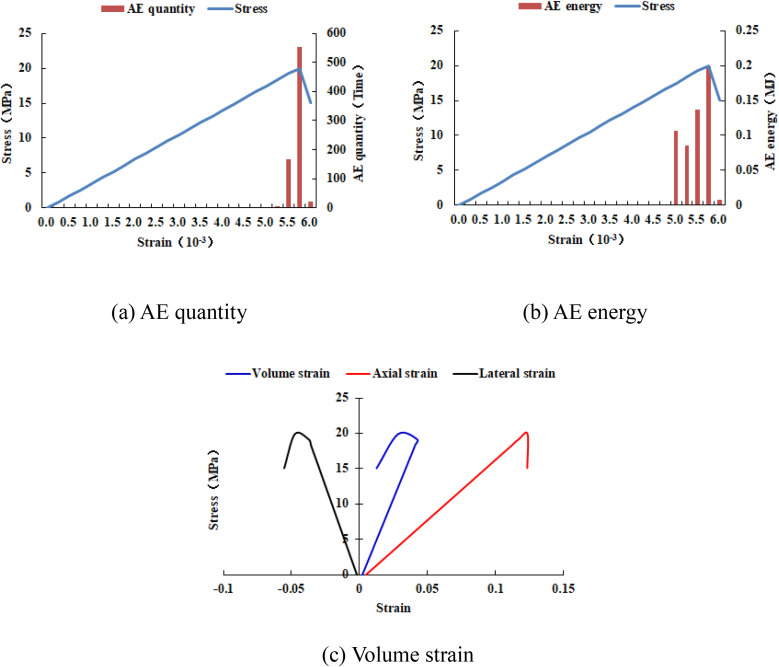
Variation trends of AE quantity, AE energy and volume strain of numerical model 40-2-EHe.

**Fig 29:**
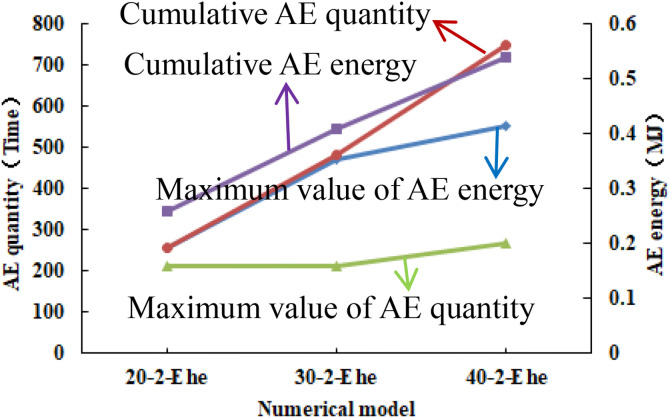
Variation trends of AE quantity and AE energy in different numerical models.

As shown in Fig 26, when the numerical model is increased to 30 rows and 30 columns, two elements of the numerical model have tensile failure. When the numerical model is increased to 40 rows and 40 columns, the entire numerical model has tensile failure and shows a significant dilatancy phenomenon, with a dilatancy capacity of 0.0217 (see Table 6). As shown in Figs 27 and 28, the maximum AE quantity and AE energy of both numerical models occur at the peak strain. When the numerical model gradually increases in size, the compressive strength of each numerical model is relatively close, and the cumulative AE quantity and cumulative AE energy increase linearly (see Fig 29). The maximum AE energy of the numerical models 20–2-EHe and 30–2-EHe is both 0.16 MJ, and that of the numerical model 40–2-EHe is 0.2 MJ.

**Table 6:** The dilatancy capacity, compressive strength, AE quantity and AE energy of each numerical model with the size of the elastic modulus heterogeneous region remaining unchanged (0.001*0.001 m) and the numerical model gradually increasing as a whole.

Numerical model	Dilatancy capacity	Compressive strength (MPa)	Maximum value of AE quantity (Time)	Cumulative AE quantity (Time)	Maximum value of AE energy (MJ)	Cumulative AE energy (MJ)
20-2-EHe	0	19.25	255	258	0.16	0.26
30-2-EHe	0.0025	19.78	470	484	0.16	0.41
40-2-EHe	0.0217	19.92	552	747	0.2	0.54

### 4.3. Heterogeneity of elastic modulus

**Fig 30:**
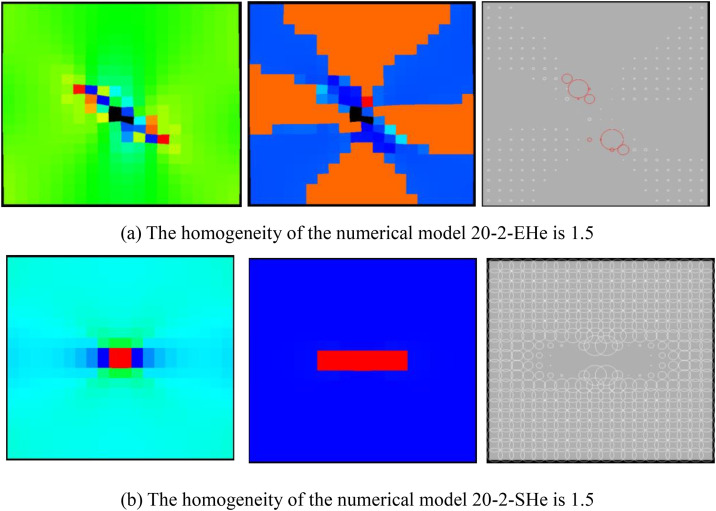
Failure Modes of different numerical models.

**Fig 31:**
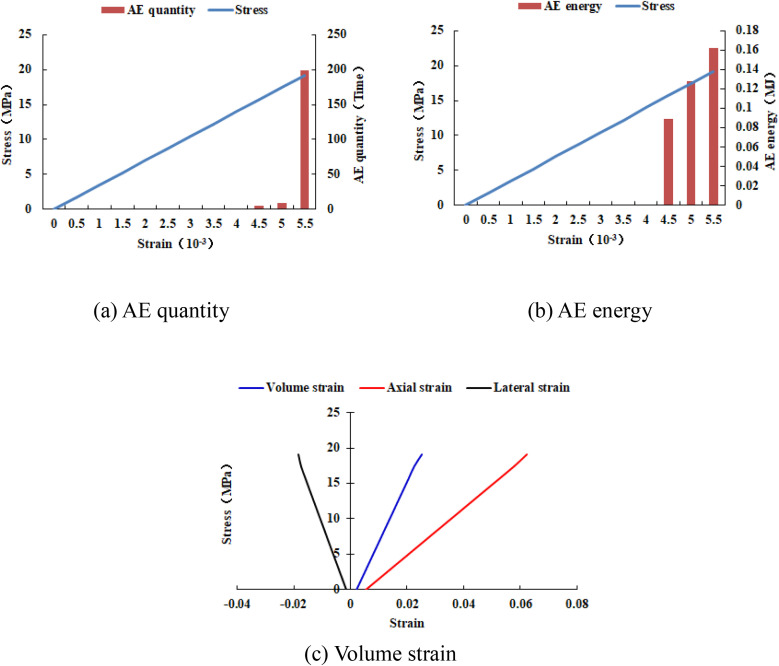
Variation trends of AE quantity, AE energy and volume strain of numerical model 20-2-EHe with a homogeneity of 1.5.

**Fig 32:**
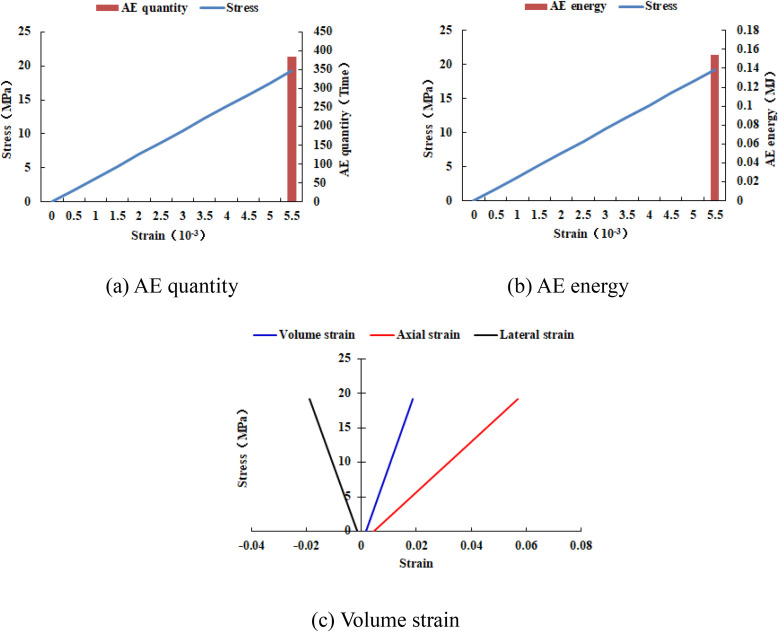
Variation trends of AE quantity, AE energy and volume strain of numerical model 20-2-SHe with a homogeneity of 1.5.

It can be seen from Figs 30 and 31 that when the homogeneity of the numerical model 20–2-EHe is 1.5, the individual elements in the numerical model have tensile failure, the maximum value of AE quantity is 199, the cumulative AE quantity is 213, the maximum value of AE energy is 0.16 MJ, and the cumulative AE energy is 0.38 MJ. When the homogeneity of the numerical model 20–2-SHe is 1.5, the numerical model is not damaged. The maximum and cumulative values of the AE quantity are both 384, and the maximum and cumulative values of the AE energy are both 0.15MJ (see Fig 32). Although neither of the two numerical models has undergone dilatancy, the AE energies of the two numerical models show different trends of change. The AE energy of the former numerical model shows a gradually increasing trend, while that of the latter numerical model is only generated at the last moment. The numerical model 20–2-EHe with a homogeneity of 1.5 has fewer deformed elements but releases more energy, indicating that the failure degree of the numerical model is more severe. The influence of the heterogeneity of the elastic modulus on the deformation failure of rocks is greater than that of compressive strength.

### 4.4. The size of the elastic modulus

**Fig 33:**
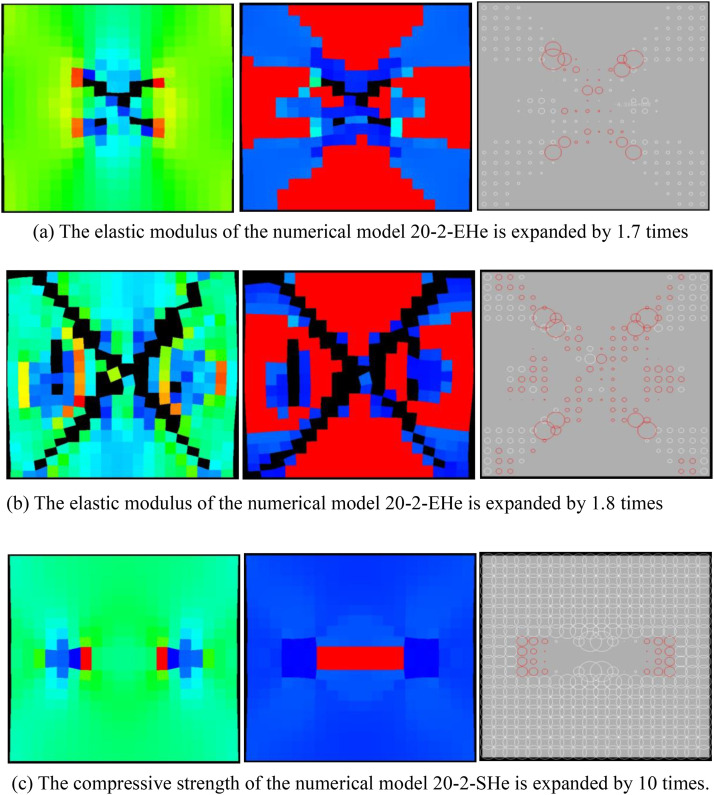
Failure mode of the numerical model.

**Fig 34:**
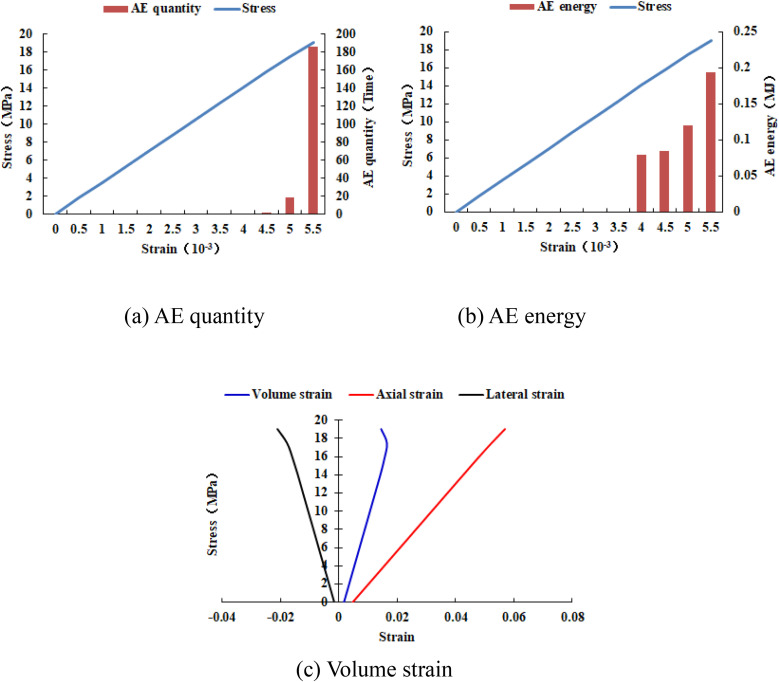
Variation trends of AE quantity, AE energy and volume strain of the numerical model 20-2-EHe when the elastic modulus is expanded by 1.7 times.

**Fig 35:**
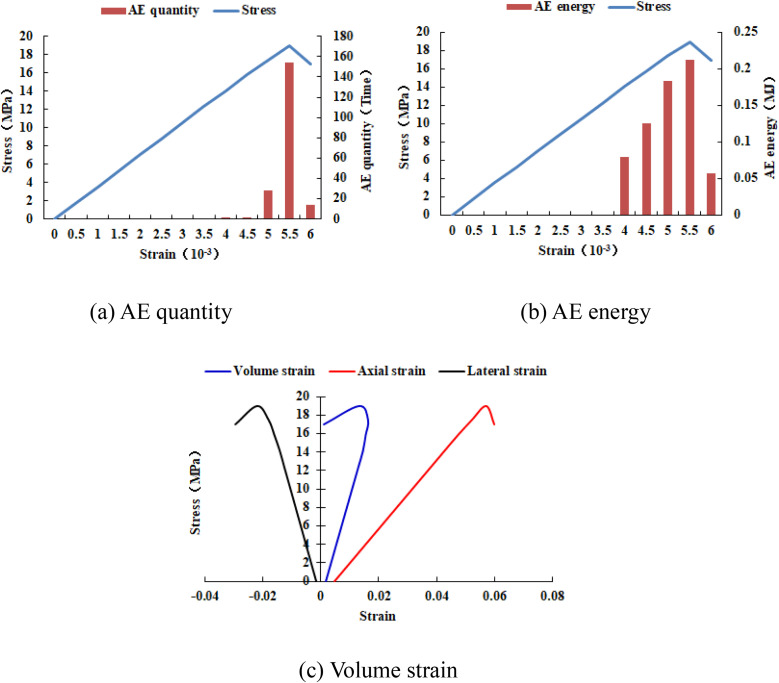
Variation trends of AE quantity, AE energy and volume strain of the numerical model 20-2-EHe when the elastic modulus is expanded by 1.8 times.

**Fig 36:**
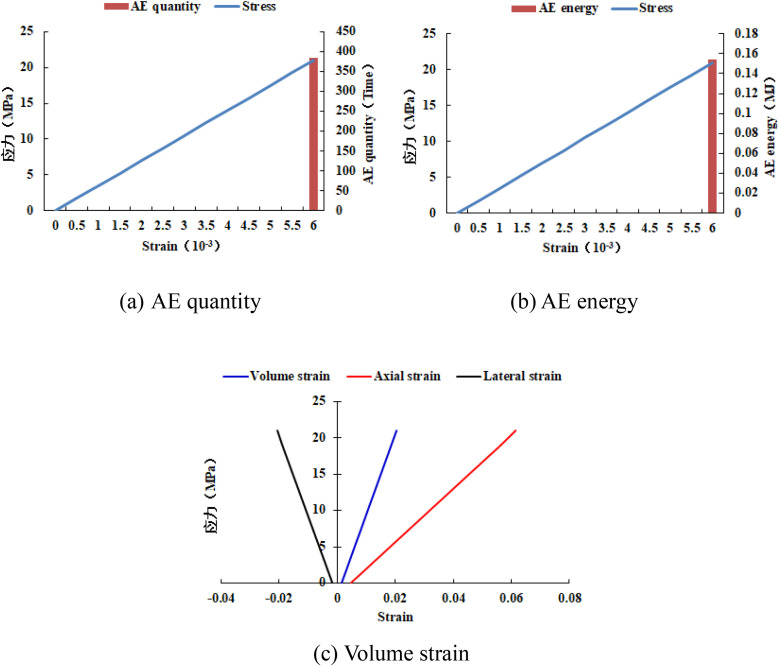
Variation trends of AE quantity, AE energy and volume strain of the numerical model 20-2-SHe when its compressive strength is expanded by 10 times.

It can be known from Figs 33–36 that when the elastic modulus of the numerical model 20–2-EHe expands by 1.7 times, the individual elements in the numerical model have tensile failure, with a dilatancy capacity of 0.0042. When the elastic modulus of the numerical model 20–2-EHe expands by 1.8 times, the numerical model produces an X-shaped tensile rupture zone with a dilatancy capacity of 0.0161. The AE quantities of the two numerical models show a sudden increase and decrease trend, while the AE energy shows a gradually increasing trend, the AE quantity, AE energy and stress all reach their maximum values simultaneously.

However, when the compressive strength of the numerical model 20–2-SHe is expanded by 10 times, the numerical model is still not damaged, and the dilatancy capacity is 0. The values and changing trends of AE quantity, AE energy and stress in this numerical model are the same as those in the numerical model 20–2-SHe with unexpanded compressive strength. The above phenomena indicate that the variation of the elastic modulus value has a greater influence on the deformation and failure of rocks than compressive strength.

## 5. Discussion

With the increase of the heterogeneous region of the elastic modulus, both the maximum value of AE quantity and the cumulative AE quantity decrease, while the maximum value of AE energy and the cumulative AE energy increase. When the heterogeneous region of the elastic modulus in the numerical model is only 4 rows and 4 columns, the numerical model is destroyed as a whole. While when the heterogeneous region of the compressive strength in the numerical model is 18 rows and 18 columns, the numerical model is destroyed as a whole. Moreover, the dilatancy capacity of 4 rows and 4 columns in the elastic modulus heterogeneous region, which is equivalent to the dilatancy capacity of the heterogeneous area of the compressive strength is 12 rows and 12 columns. The maximum value of AE quantity, the cumulative AE quantity, the maximum value of AE energy and the cumulative AE energy of the heterogeneous area of the elastic modulus is 4 rows and 4 columns are equivalent to each value of the heterogeneous area of the compressive strength is 10 rows and 10 columns.

When the heterogeneous region of the elastic modulus is 2 rows and 2 columns and the overall numerical model is 40 rows and 40 columns, the numerical model is damaged. When the heterogeneous regions of compressive strength are all 2 rows and 2 columns and the overall numerical model is 60 rows and 60 columns, the numerical model is damaged. The maximum value of AE energy and the cumulative AE energy of the two numerical models are basically the same, and the fracture zones are both “X-shaped”. The dilatancy capacity of the numerical model 40–2-EHe is equivalent to that of the numerical model 50–2-SHe.

The parameters such as failure mode, dilatancy capacity, AE quantity and AE energy of the model all indicate that the heterogeneous region of the elastic modulus has a much greater influence on the deformation failure of the numerical model than the heterogeneous region of the compressive strength.

When the homogeneity of the numerical model 20–2-She compressive strength heterogeneous region and the numerical model 20–2-Ehe elastic modulus heterogeneous region is 3, the numerical model is not destroyed. When the homogeneity of the two numerical models is reduced to 1.5, the individual elements of the numerical model 20–2-Ehe are destroyed, and the numerical model 20–2-She is still not destroyed. When the elastic modulus of the numerical model 20–2-EHe is expanded by 1.8 times, the numerical model produces an X-shaped tensile rupture zone with a dilatancy capacity of 0.0161. When the compressive strength of the numerical model 20–2-SHe is expanded by 10 times, the numerical model is still not damaged, and the dilatancy capacity is 0. All the above phenomena indicate that the magnitude and heterogeneity of the elastic modulus have a much greater impact on the deformation and failure of the numerical model than the magnitude and heterogeneity of the compressive strength.

## 6. Conclusion

(1) The greater the proportion of heterogeneous regions in compressive strength and elastic modulus, the less AE quantity is released when the rock sample is damaged, while the greater the AE energy released, that is, the more severe the rock damage. The failure of heterogeneous regions in compressive strength and elastic modulus is the reason for the significant increase in the dilatancy capacity of rock samples.
(2) The numerical model 20–2-SHe with a homogeneity of 3, a homogeneity of 1.5, and a compressive strength expanded by 10 times has the same AE quantity, AE energy and compressive strength, and none of the numerical models have been damaged, indicating that the deformation and damage of the numerical model with a smaller heterogeneous area of compressive strength is not affected by homogeneity and compressive strength.
(3) The dilatancy capacity of the numerical model 20–4-EHe is equivalent to that of the numerical model 20–12-SHe. The numerical model 20–2-EHe with an elastic modulus increase of 1.8 times suffers overall failure. The numerical model 20–2-EHe with an elastic modulus homogeneity decrease of 1 time has internal element failure. All the above shows that the deformation and failure of rock is more susceptible to the change of elastic modulus.
(4) When the heterogeneous area of compressive strength increased from 16 rows and 16 columns (local failure of the numerical model) to 18 rows and 18 columns (overall failure of the numerical model), the dilatancy capacity increased from 0.0137 to 0.0706, increasing by 5.2 times; When the expansion factor of the elastic modulus increases from 1.7 (local failure of the numerical model) to 1.8 (overall failure of the numerical model), the dilatancy capacity increases from 0.0042 to 0.0161, increasing by 3.8 times. This phenomenon also exists in the other groups of numerical simulations, that is, the numerical model expands from local failure to overall failure, and the dilatancy capacity suddenly increases. Moreover, in each group of numerical simulations with different variables, the greater the cumulative AE energy, the greater the dilatancy capacity.
